# Mapping forests in monsoon Asia with ALOS PALSAR 50-m mosaic images and MODIS imagery in 2010

**DOI:** 10.1038/srep20880

**Published:** 2016-02-11

**Authors:** Yuanwei Qin, Xiangming Xiao, Jinwei Dong, Geli Zhang, Partha Sarathi Roy, Pawan Kumar Joshi, Hammad Gilani, Manchiraju Sri Ramachandra Murthy, Cui Jin, Jie Wang, Yao Zhang, Bangqian Chen, Michael Angelo Menarguez, Chandrashekhar M. Biradar, Rajen Bajgain, Xiangping Li, Shengqi Dai, Ying Hou, Fengfei Xin, Berrien Moore III

**Affiliations:** 1 Department of Microbiology and Plant Biology, Center for Spatial Analysis, University of Oklahoma, Norman, OK, 73019, USA; 2 Institute of Biodiversity Science, Fudan University, Shanghai, 200433, China; 3 University Center for Earth and Space Science, University of Hyderabad, Hyderabad, 500046, India; 4 School of Environmental Sciences, Jawaharlal Nehru University, New Delhi, 110067, India; 5 International Centre for Integrated Mountain Development, Kathmandu, 44700, Nepal; 6 Danzhou Investigation & Experiment Station of Tropical Cops, Ministry of Agriculture, Rubber Research Institute, Chinese Academy of Tropical Agricultural Sciences, Danzhou 571737, China; 7 International Center for Agricultural Research in the Dry Areas, Amman, 11195, Jordan; 8 College of Atmospheric and Geographic Science, University of Oklahoma, Norman, OK, 73019, USA

## Abstract

Extensive forest changes have occurred in monsoon Asia, substantially affecting climate, carbon cycle and biodiversity. Accurate forest cover maps at fine spatial resolutions are required to qualify and quantify these effects. In this study, an algorithm was developed to map forests in 2010, with the use of structure and biomass information from the Advanced Land Observation System (ALOS) Phased Array L-band Synthetic Aperture Radar (PALSAR) mosaic dataset and the phenological information from MODerate Resolution Imaging Spectroradiometer (MOD13Q1 and MOD09A1) products. Our forest map (PALSARMOD50 m F/NF) was assessed through randomly selected ground truth samples from high spatial resolution images and had an overall accuracy of 95%. Total area of forests in monsoon Asia in 2010 was estimated to be ~6.3 × 10^6 ^km^2^. The distribution of evergreen and deciduous forests agreed reasonably well with the median Normalized Difference Vegetation Index (NDVI) in winter. PALSARMOD50 m F/NF map showed good spatial and areal agreements with selected forest maps generated by the Japan Aerospace Exploration Agency (JAXA F/NF), European Space Agency (ESA F/NF), Boston University (MCD12Q1 F/NF), Food and Agricultural Organization (FAO FRA), and University of Maryland (Landsat forests), but relatively large differences and uncertainties in tropical forests and evergreen and deciduous forests.

Human activities in monsoon Asia (see Supplementary Fig. S1) have resulted in intensive land use and cover changes, especially for forests, which have considerable effects on climate change, biodiversity, and ecosystem services^1^,^2^,^3^. Forest changes, especially in tropical regions, are regarded as a major source of greenhouse gas (GHG) emissions. Deforestation and forest degradation are responsible for 12–20% of global GHG emissions per year^3^,^4^. To mitigate future global climate change in a cost effective way, the REDD+ (Reduce Emissions from Deforestation and Forest Degradation) mechanism under the United Nations Framework Convention on Climate Change (UNFCCC) was proposed (http://unfccc.int/2860.php) with the aims of reducing emissions from deforestation and forest degradation, as well as promoting forest conservation, sustainable management of forests, and enhancement of forest carbon stocks in developing countries. Accurate and consistent detection of forest changes at fine spatial resolutions is required for the estimation of GHG emissions and for developing polices to reduce deforestation and forest degradation^5^,^6^.

Although much attention has been given to forest protection, large areas of forests are diminishing in tropical regions in monsoon Asia (see Supplementary Fig. S2), as highly dense, poor populations depend on forests and other local natural ecosystems^7^. Agricultural land expansion via deforestation, and the replacement of primary forests by forest plantations (e.g., rubber, eucalyptus, oil palm) or cash crops are common and have resulted in tremendous losses of forest cover, biodiversity, carbon storage and sustainability^1^,^7^,^8^,^9^. For example, the forest cover loss in Indonesia over the past 20 years has been high and continually increasing^9^, particularly in areas with a high proportion of primary forests in protection areas; the annual loss of annual primary forest cover was higher than that in Brazil during 2000–2012^8^. National-scale reforestation and afforestation is considered an important contributor to East Asia’s becoming a high carbon dioxide (CO_2_) uptake region^10^,^11^. For example, annual forest cover increased about 2.5 × 10^4 ^km^2^ in China during 1990–2012 (see Supplementary Fig. S2).

Multiple historical forest cover datasets were developed with reasonably good accuracies using different satellite images such as 8-km or 1-km Advanced Very High Resolution Radiometer (AVHRR)^12^,^13^, 500-m Moderate Resolution Imaging Spectroradiometer (MODIS)^14^,^15^, 30-m Landsat^16^,^17^, and 25-m or 50-m Phased Array L-band Synthetic Aperture Radar (PALSAR)^18^,^19^. Optical remote sensing is sensitive to the canopy structure of vegetation, which may overestimate or omit the extent of the woody vegetation in some cases^18^. The L-band PALSAR images, independent of clouds and day/night, show promising potential for global forest mapping^18^. Optical images contain information on the reflectance of the land surface, while radar images contain information on the structure, biomass, and dielectric of the land surface. Land cover types inseparable by optical images may be distinguished by radar images due to the complementary information provided by these two kinds of images. Recent studies investigated the advantages of the integration of optical and radar images to identify land cover types^20^,^21^,^22^, and their results showed that the integration of optical and radar images can achieve higher classification accuracy than those generated by an individual sensor.

The objectives of this study are to (1) map the forests (both evergreen and deciduous) cover at the spatial resolution of 50 m in monsoon Asia in 2010 using a decision tree algorithm and the PALSAR and MODIS data; and (2) investigate the uncertainties between our resultant forest/non-forest maps (PALSARMOD50m F/NF, ALOS PALSAR- and MODIS-based forest/non-forest maps) and the selected forest cover datasets (see Supplementary Table S1). The selected forest datasets are currently the best available products for 2010, including the 50-m forest/non-forest map generated by PALSAR mosaic datasets from the Japan Aerospace Exploration Agency (JAXA F/NF), 300-m forest/non-forest map generated by Medium Resolution Imaging Spectrometer (MERIS) and SPOT VEGETATION from the Europe Space Agency (ESA F/NF), 500-m forest/non-forest map from MCD12Q1 (MCD12Q1 F/NF), forest area statistics in countries from the Food and Agriculture Organization Global Forest Resources Assessment (FAO FRA), and Landsat-based forest maps produced by researchers in the University of Maryland. This study aims to provide a simple and effective algorithm for monitoring forests in monsoon Asia at a fine spatial resolution, and a baseline forest cover map for investigating forest dynamics and their effects on GHG emissions, carbon stock and ecosystems, which is important for the success of REDD+.

## Results

### PALSARMOD50m forest/non-forest map in 2010 and accuracy assessment

**Figure 1:**
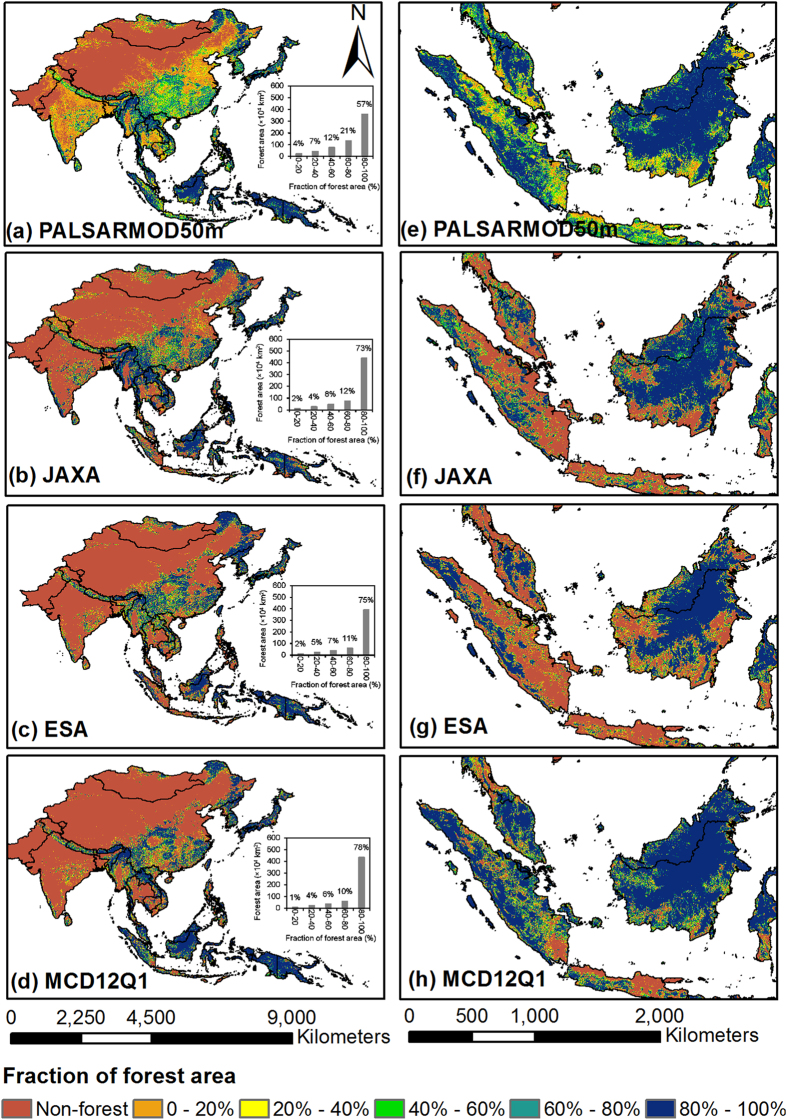
Spatial distribution of forests in monsoon Asia at the spatial resolution of 1,500 m in 2010 from multiple forest/non-forest maps. (**a**) PALSARMOD50m forest/non-forest map, produced by the developed algorithm in this study. (**b**) JAXA forest/non-forest map, provided by the Earth Observation Research Center, JAXA (ftp://ftp.eorc.jaxa.jp/pub/ALOS-2/PALSAR_MSC/50m_MSC). (**c**) ESA forest/non-forest map, provided by ESA Climate Change Initiative-Land Cover (CCI-LC) project (http://www.esa-landcover-cci.org/?q=node/158). (**d**) MCD12Q1 forest/non-forest map, derived from MODIS/Terra + Aqua Land Cover Type Yearly L3 Global 500m SIN Grid V051 product, provided by Earth Observing System Data and Information System, National Aeronautics and Space Administration (http://reverb.echo.nasa.gov/reverb). (**a–d**) were aggregated into 1,500 m for spatial comparison in ArcGIS 10.1. (**e–h**) Zoomed-in forest/non-forest maps in Southeast Asia from (**a–d**), respectively. This figure was produced using ArcGIS 10.1.

Extensive forests were mainly distributed in Southeast Asia, northeastern and southern China, the Korean Peninsula, and Japan, while sparse forests were mainly in South Asia, western and northern China, and Mongolia dominated by cropland, prairie, or desert (Fig. 1(a)). The total forest area in monsoon Asia (23 countries) in 2010 was estimated to be ~632.4 × 10^4 ^km^2^, and China and Indonesia covered the largest proportions, approximately 29.6% and 22.2%, respectively. The resultant PALSARMOD50m F/NF map at 50-m resolution was assessed through a confusion matrix based on randomly selected forest/non-forest Area of Interests (AOIs) from high spatial resolution images in Google Earth. The Kappa coefficient, Overall Accuracy, User Accuracy and Producer Accuracy of PALSARMOD50m F/NF were approximately 0.90, 95.9%, 98.9%, and 88.4%, respectively (see Supplementary Table S2).

### PALSARMOD50m evergreen and deciduous forest maps

**Figure 2:**
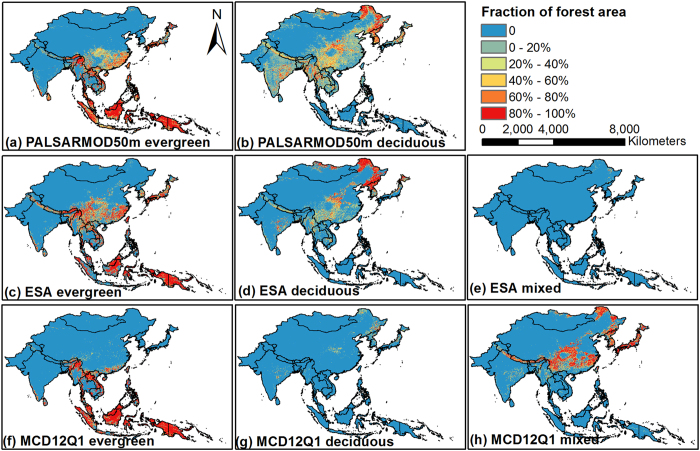
Spatial distribution of evergreen, deciduous, and mixed forests from PALSARMOD50m, ESA, and MCD12Q1 forest maps at the spatial resolution of 1,500 m in monsoon Asia in 2010. (**a**) PALSARMOD50m evergreen forests and (**b**) PALSARMOD50m deciduous forests, produced by the developed algorithm in this study. (**c**) ESA evergreen forests, (**d**) ESA deciduous forests, and (**e**) ESA mixed forests, provided by ESA CCI-LC project (http://www.esa-landcover-cci.org/?q=node/158). (**f**) MCD12Q1 evergreen forests, (**g**) MCD12Q1 deciduous forests, and (**h**) MCD12Q1 mixed forests, derived from MODIS/Terra + Aqua Land Cover Type Yearly L3 Global 500m SIN Grid V051 product, provided by Earth Observing System Data and Information System, National Aeronautics and Space Administration (http://reverb.echo.nasa.gov/reverb). All these maps were aggregated into 1,500 m for spatial comparison in ArcGIS 10.1. This figure was produced using ArcGIS 10.1.

**Figure 3:**
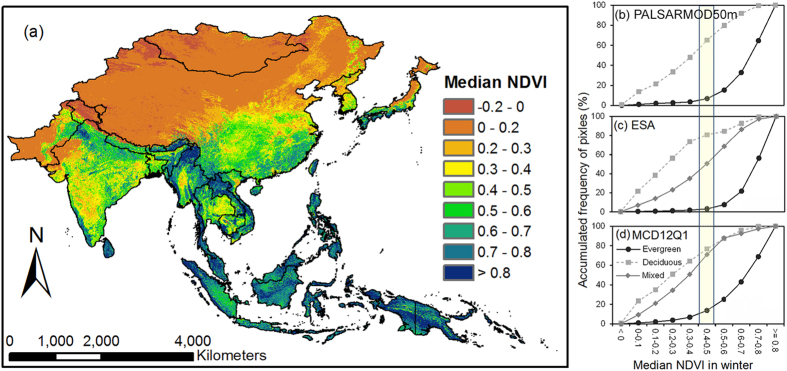
The median NDVI of forests in winter (December, January, and February) in monsoon Asia from 2000 to 2014. (**a**) The distribution map of median NDVI in winter from MOD13Q1 product, at the spatial resolution of 250 m, derived from MODIS/Terra Vegetation Indices 16-Day L3 Global 250m SIN Grid V005 product, downloaded from Earth Observing System Data and Information System, National Aeronautics and Space Administration (http://reverb.echo.nasa.gov/reverb). (**b**–**d**) are the median NDVI distribution of PALSARMOD50m, ESA, and MODIS forests, respectively. This figure was produced using ArcGIS 10.1.

The areas of evergreen and deciduous forests were estimated to be ~398.4 × 10^4 ^km^2^ (63.0% of the total forest area) and ~233.8 × 10^4 ^km^2^ (37.0%) in monsoon Asia in 2010, respectively. The spatial distribution of both evergreen and deciduous forests showed obvious regional characteristics (Fig. 2(a,b)). Evergreen forests were mainly distributed in tropical regions, *i.e.*, Southeast Asia, southern China, and Japan, while deciduous forests were mainly in the north temperate zone (Northeast Asia and southwestern China), and the subtropical zone with its obvious dry season (Indo-China and India). The reasonability of the resultant evergreen and deciduous forests were evaluated using a distribution map of median NDVI (Fig. 3(a)) in winter (December, January, and February) generated by good observations without cloud cover, shadow, or snow/ice from MOD13Q1 product. Approximately 93.2% and 65.1% of evergreen and deciduous forest pixels, respectively, had median NDVI values higher or lower than 0.5 in the PALSARMOD50m F/NF map (Fig. 3(b)).

### Spatial and area comparisons of multiple forest cover datasets

**Figure 4:**
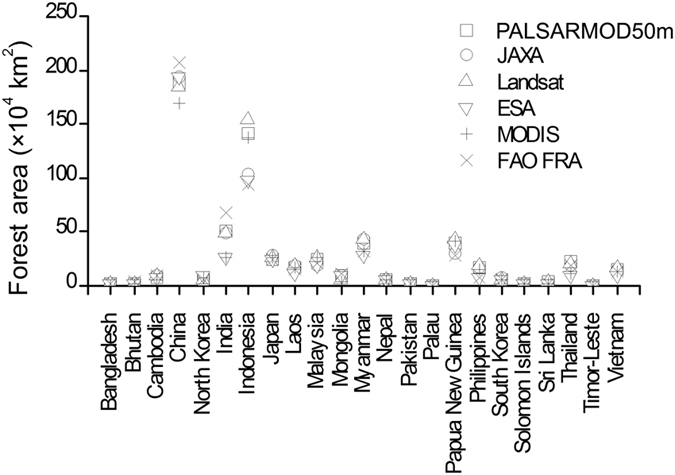
Forest area comparison from multiple forest datasets at the country scale in monsoon Asia in 2010. This figure was produced using Origin 8.0.

Geographically, PALSARMOD50m, JAXA, ESA, and MCD12Q1 F/NF maps presented similar spatial distributions of forests in monsoon Asia in 2010 (Fig. 1(a–d)). The fraction of forest area agreed reasonably well between PALSARMOD50m F/NF map and JAXA, ESA, and MCD12Q1 F/NF maps at the spatial resolution of 1,500 m, and the differences of over 80% pixels were in the range of ±25% (see Supplementary Fig. S3(a–c)). PALSARMOD50m F/NF identified more forests than the JAXA and ESA F/NF maps in tropical regions (Fig. 1(e–h) and Supplementary Fig. S3(d–f)). For the area comparison, the forest area of PALSARMOD50m F/NF was very close to that estimated by the Landsat-based forest map^16^, and higher than that of the other forest datasets in Monsoon Asia (see Supplementary Fig. S4(a)). The forest areas from PALSARMOD50m F/NF were very close to those derived from Landsat images, and differed considerably from the other forest datasets, especially for China, Indonesia and India (Fig. 4, Supplementary Fig. S4 and Table S3).

### Spatial and area comparisons of evergreen and deciduous forest maps

**Figure 5:**
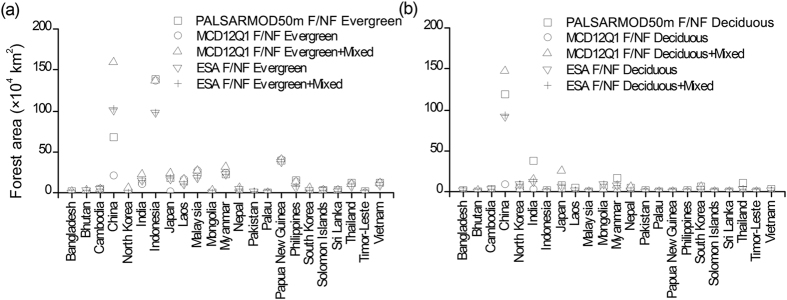
Area comparisons of (**a**) evergreen and (**b**) deciduous forests from multiple forest datasets at the country scale in monsoon Asia in 2010. This figure was produced using Origin 8.0.

We compared our resultant evergreen and deciduous forest maps with ESA and MCD12Q1 F/NF maps, as only two out of the selected forest maps had evergreen and deciduous forest classes. In this study, PALSARMOD50m evergreen forest was compared with evergreen and (evergreen + mixed forests) from ESA and MCD12Q1 F/NF maps, respectively; PALSARMOD50m deciduous forest was compared with deciduous and (deciduous + mixed forests) from ESA and MCD12Q1 F/NF maps, respectively. The results showed that large uncertainties existed among PALSARMOD50m, ESA, and MCD12Q1 evergreen and deciduous forest maps (Figs 2 and 5, Supplementary Table. S3), and their differences were over ±50% in some disputed areas (see Supplementary Fig. S5). As evergreen forests dominate the tropical regions, good agreement between evergreen and deciduous forests were achieved for PALSARMOD50m F/NF and ESA, MCD12Q1 F/NF maps, except for the differences contributed by the forest baseline maps. However, large differences of evergreen and deciduous forests existed in other areas (e.g., China, South Korea), and the mixed pixels of evergreen and deciduous forests might be the reason.

## Discussion

The algorithm developed in this study presented the potential for large areas of forest (evergreen and deciduous) mapping using uniform thresholds of PALSAR backscatter coefficients, NDVI, and Land Surface Water Index (LSWI). PALSAR images could reduce the limitation of frequent clouds on forest mapping with optical images and could exclude other evergreen vegetation cover types such as evergreen shrubs and continuous crops that were difficult to distinguish with optical remote sensing. NDVI can eliminate the commission error of forests caused by mountains with complex reflectance/backscatter environment (e.g. the Qinghai-Tibetan Plateau and urban area), and LSWI is an effective indicator to identify evergreen and deciduous forests based on their phenological differences^23^,^24^. The resultant 50-m PALSARMOD50m F/NF map was assessed with reasonably high accuracy and presented good spatial and areal agreement with the selected forest datasets in monsoon Asia. The PALSARMOD50m evergreen and deciduous forests were assessed, yielding good reasonability based on the median NDVI in winter. As shown in Fig. 3, a certain number of mixed pixels may exist in these evergreen and deciduous forests maps arising from multiple sources, particularly for deciduous forests. Two factors are responsible for this. First, evergreen forests in the temperate and subtropical climate regions without snow in the winter have high NDVI values, while deciduous forests in high latitude regions show negative bias in NDVI values as a result of snow cover, which makes identifying evergreen and deciduous forests difficult^25^. Second, in the tropical monsoon climate regions (e.g. India, Indo-China), some trees usually defoliate in the dry season (March, April, and May), which could be investigated by LSWI^23^ rather than the median NDVI value in winter.

Several factors might affect the accuracy of our forest (evergreen and deciduous) map. First, the forest definition varies among the map producers. Mature forests were easily identified from high spatial resolution images for algorithm training and accuracy assessment through their colors, intensity, and structure; however, the sparse and/or low-height forests were difficult to be identified by the interpreters. Second, PALSAR data in the growing season was preferred for forest mapping, but a small number of PALSAR datasets from outside the growing season were also included in the JAXA PALSAR 50-m mosaic images, which affected the forest mapping results in the boreal area. Third, the mixed pixel was identified as either evergreen or deciduous forest with stronger signals.

Large differences and uncertainties in forest distribution among the multi-source forest maps were observed in the tropical regions of monsoon Asia (see Supplementary Fig. S6). Several factors may contribute to these uncertainties. First, forest definitions are different in these forest cover datasets: forest coverage varies from >10% to >60% and and tree height varies from >2-m to >5-m (see Supplementary Table S1). The difference in forest definition was reported to be the major reason causing uncertainties of forest cover area estimation^26^. Secondly, different ground truth samples were used in those datasets. Thirdly, different forest mapping algorithms were used in those datasets. Thus, the use of similar forest definitions and the share of training samples would help reduce the uncertainties of forest cover estimation. High cloud frequency (over 90%) occurs in Southeast Asia, southwestern China, and Central Asia, which limits the data availability of optical images for tracking forest changes in these regions^27^. Therefore, multi-year optical images, which were usually used to generate these forest maps^14^,^16^,^17^,^28^, could miss annual forest changes in some hotspots with extensive deforestation and/or reforestation/afforestation. The coarse spatial resolution images (e.g., 300-m MEdium Resolution Imaging Spectrometer (MERIS), 500-m MODIS, and 1-km SPOT VEGETATION) can track the dynamics of large-size intact forests, but the complicated and fragmented landscape contributed by selective logging of timber was hard to monitor^29^,^30^,^31^. Illegal deforestation and forest exports were common in Southeast Asia^32^,^33^,^34^,^35^; for example, approximately half of Vietnam’s wood imports (approximately 39% of the regrowth of Vietnam’s forests) during 1987–2006 was illegal^35^. However, the official statistics seemed to struggle to track illegal logging and thus may easily have large uncertainties in the FAO FRA data^8^.

Forest distribution and its dynamics are important input datasets for models to investigate the interactions between land cover, climate, and ecosystems^1^,^2^,^36^,^37^. The great loss of humid tropical forests, especially the burning and oxidation of peat swamp forests, contributed a large proportion of total CO_2_ emissions and endanger the biodiversity^3^,^38^,^39^ in Southeast Asia. The differences in vegetation cover, seasonal albedo, surface roughness, and fluxes of water, energy and CO_2_^40^ caused by phenology and growing characteristics, as well as spatio-temporal changes of evergreen and deciduous forests can help to investigate their biophysical and biochemical effects on climate cooling or warming, carbon cycle, and biodiversity^34^. Moreover, the dynamics of evergreen and deciduous forests can illustrate their responses to different disturbances, e.g., the expansion of deciduous forests into evergreen forests in boreal region as a symptom of climate warming^2^ or the deciduous rubber plantation expansion into primary evergreen forests in the northern tropical region^41^.

## Methods

**Figure 6:**
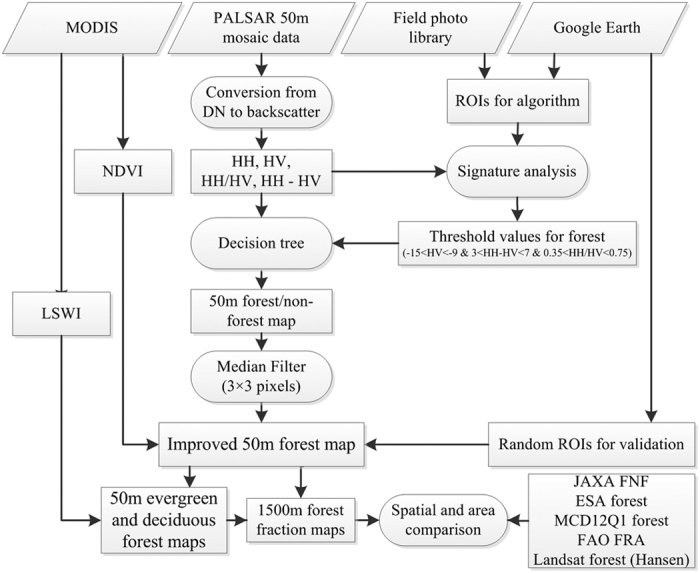
Workflow of forest mapping in monsoon Asia using 50-m PALSAR, 250-m MOD13Q1 NDVI, and 500-m LSWI derived from the MOD09A1 product. This figure was produced using Microsoft Visio 2010.

The workflow of this study is composed of five parts as the followings (Fig. 6): (1) image collection and preprocessing, (2) algorithm development, (3) algorithm implementation, (4) accuracy assessment; and (5) comparison with other available forest cover maps.

### Study area

Monsoon Asia covers a large area in East Asia (China, Mongolia, North Korea, South Korea, and Japan), Southeast Asia (Myanmar, Thailand, Laos, Cambodia, Vietnam, Philippines, Brunei, Malaysia, Singapore, Indonesia, Palau, Timor-Leste, Papua New Guinea, and Solomon Islands), and South Asia (India, Bhutan, Bangladesh, Nepal, Pakistan, and Sri Lanka). Singapore is not included in this study, as its forest area is not available from the online Landsat-based forest maps produced by the University of Maryland. The land area of monsoon Asia is about 16% of the world land area and, in 2010, supported about 54% of the world population^42^. Monsoon Asia is a region with great interaction of land and ocean. The climate has distinct regional and seasonal characteristics due to the Asian monsoons and complex topography (see Supplementary Fig. S1). Tropical, subtropical, and temperate zones comprise southern to northern monsoon Asia. The marine monsoon prevails from May to September and brings a large amount of rainfall, and the continental monsoons occurring from November to March bring cool, dry air. Southeast Asia is hot and rainy all year round, dominated by a tropical rainforest climate.

Elevation in the region varies from −156 m to 8,685 m (see Supplementary Fig. S1). The Qinghai-Tibetan Plateau has an average elevation of approximately 4,500 m, followed by the plateaus and mountains (~1,000 m) of the Mongolia Plateau and Indo-China, and the plains and low hills (<500 m) in coastal areas. The area of forest is the second largest land cover type in monsoon Asia, about 28% of the land area, second only to the area of agricultural land^42^.

### PALSAR 50-m orthorectified image data

The 50-m PALSAR orthorectified mosaic data at fine beam dual polarization mode for 2010 is aggregated from the original observation with minimum response to surface moisture^18^, which is available from the Earth Observation Research Center, JAXA (ftp://ftp.eorc.jaxa.jp/pub/ALOS-2/PALSAR_MSC/50m_MSC). PALSAR HH and HV backscatter data are slope corrected and orthorectified with a geometric accuracy of about 12 m, using the 90-m Shuttle Radar Topography Mission (SRTM) Digital Elevation Model (DEM), and radiometrically calibrated. The Digital Number (DN) values (amplitude values) were converted into gamma-naught backscattering coefficients in decibels (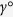) using a calibration coefficient, which were used for forest/non-forest mapping in monsoon Asia.


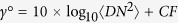


where CF is the absolute calibration factor of −83^43^.

### MODIS image data

MOD09A1, MOD13Q1 and MCD12Q1 products, derived from daily MODIS observations available from National Aeronautics and Space Administration (NASA) Earth Observing System Data and Information System (http://reverb.echo.nasa.gov/reverb/), were used in this study. MOD09A1 provides MODIS bands 1–7 surface reflectance products with quality assessment information every 8-day cycle at the spatial resolution of 500 m from 2000 to date^44^. These 7 bands, in the visible and short-wave infrared (SWIR) spectrum, are designed primarily for land, cloud, and aerosol use. LSWI is a satellite-derived index from the near infrared (NIR) and SWIR bands from MOD09A1 product. The SWIR band is sensitive to the total amount of liquid water from leaves and their soil background^45^,^46^. LSWI is sensitive to equivalent water thickness^47^, and can be used as an indicator to identify evergreen and deciduous forests^23^,^24^.


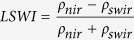


where the 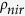 and 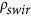 are the land surface reflectance of NIR (841–875 nm) and SWIR (1628–1652 nm) bands, respectively, from MOD09A1 product at the spatial resolution of 500 m.

MOD13Q1 has provided NDVI, EVI, and their quality assessment information every 16 days at the spatial resolution of 250 m from 2000 to date, which can be used for global monitoring of vegetation conditions. The annual MCD12Q1 includes five different land cover classification systems at the spatial resolution of 500 m^14^. The forest classes in the International Geosphere-Biosphere Programme (IGBP) classification system were used to compare our results: evergreen needleleaf forest, evergreen broadleaf forest, deciduous needleleaf forest, deciduous broadleaf forest, and mixed forest.

### Algorithms for the forest and non-forest map at 50-m resolution

Forest is defined in the FAO as land with tree canopy cover greater than 10%^48^. We used the decision classification algorithm to map forests with the updated thresholds derived from ground truth samples of different land cover types (forests, cropland, water, and built-up) in mainland Southeast Asia^19^,^30^. Forests, cropland, water, and built-up lands present different characteristics of two polarizations (HH and HV), HH/HV (Ratio), and HH-HV (Difference), indicating the potential of their combination to identify these land cover types (see Supplementary Fig. S7). First, water can be identified easily as it has very low HH and HV values. Second, forests have high HH and HV values, and low Difference values, although these partly overlap with built-up lands. Third, most cropland can also be identified, although it may partly overlap with water. L-band PALSAR data can retrieve the structure and above ground biomass (AGB) of forests^49^,^50^, as it possesses great penetration into forests, substantial volume scattering through the incident energy interaction with large trunks and branch components. Forests, especially for the mature forests, usually have dense and large canopy and relative high AGB from tremendous number of leaves, branches, stems, and trunks. Recent studies show that forests and forest AGB exhibit a certain range of PALSAR backscattering coefficients, respectively^50^,^51^,^52^. Therefore, the uniform thresholds were used for forest mapping: −15 < HV < −9 & 3 < Difference < 7 & 0.35 < Ratio < 0.75. Finally, the land cover mapping results were merged into forest and non-forest. A median filter (3 × 3 pixels) was then applied to recode isolated pixels classified differently than the majority class of the window due to image noise^18^,^53^.

Maximum NDVI (NDVI_max_) from MOD13Q1 in 2010 was used to reduce the potential commission error from sparsely vegetated land with complex structures and rough land surfaces (e.g., rock mountains, large buildings), which had high PALSAR backscatter values similar to forests. Forests usually have an NDVI_max_ above 0.5, while the NDVI_max_ of sparsely vegetated lands (e.g., rocks, desert, buildings) is lower than 0.3, based on our statistics (see Supplementary Fig. S8) and the previous studies^54^,^55^,^56^,^57^. The growing season defined by the period of night Land Surface Temperature >0 °C was produced from MOD11A2 product. We then generated a vegetation cover using the 16-day composite MOD13Q1 NDVI product during the growing season for 2010^58^, after excluding bad observations (cloud, cloud shadow, and snow/ice), and based on the threshold value of NDVI_max_ greater than or equal to 0.5 (see Supplementary Fig. S9).

### Algorithms for evergreen and deciduous vegetation maps derived from phenological analysis of MODIS vegetation indices at 250-m and 500-m resolutions

The seasonal profiles analysis of NDVI, EVI, and LSWI provides the basis for distinguishing evergreen and deciduous forests (see Supplementary Fig. S10). Green leaves yield high NDVI values (~0.8) and LSWI >0 all year around, but senescent plants (senescent leaves and branches) in winter and/or dry season have low NDVI values (<0.4) and LSWI <0. Pixels fitting the criteria of LSWI >0 for all the good quality images in 2010 were identified as evergreen cover^23^, and the other pixels as deciduous cover (see Supplementary Fig. S11).

The evergreen vegetation usually has green leaves in winter, while deciduous vegetation does not. The median NDVIs out of MOD13Q1 NDVI at the spatial resolution of 250 m in winter (December, January, and February) from 2000 to 2014 were used to assess the reasonability of the produced evergreen and deciduous forests maps.

### Evergreen and deciduous forest map at 50-m resolution

To map evergreen and deciduous forests, we developed an algorithm by the integration of strong points from our previous studies^19^,^23^, *i.e.*, using the structure and biomass information to extract forest distribution and using the phenology of forests to distinguish evergreen and deciduous forests. In this study, we overlaid our 50-m PALSAR-based forest/non-forest map with a 500-m evergreen/deciduous cover map to obtain the 50-m evergreen and deciduous forests map in monsoon Asia in 2010.

### Accuracy assessment of forest map

**Figure 7:**
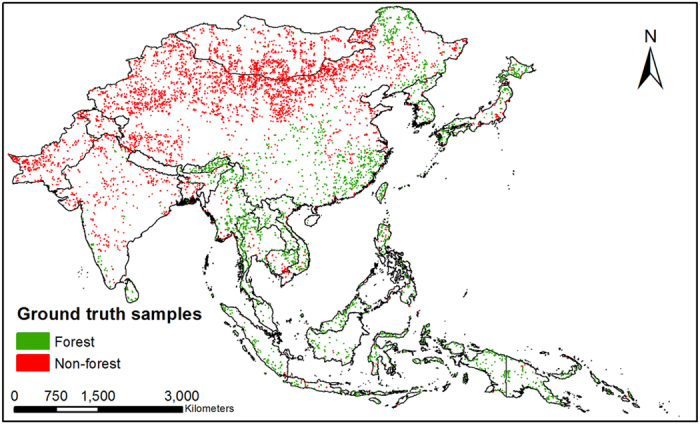
Ground truth samples for the accuracy assessment of PALSARMOD50m forest/non forest map, generated by a random sampling method in IDL 8.4. This figure was produced using ArcGIS 10.1.

A random sampling method was developed to generate a large number of ground truth samples to assess the accuracy of our forest mapping results. First, we randomly generated 20 pixels at the spatial resolution of 500 m (MODIS pixel size) in each 1 × 1 degree tile using the IDL 8.4 random function. The files were organized in 5-degree latitude-longitude geographical unit in kmz file format. Second, we opened those kmz files, overlaid them on the high spatial resolution images in Google Earth, and distinguished forest and non-forest pixels. If a pixel is covered by 90% or more forests, this pixel will be identified as forest; the same criteria applies for non-forest selection. The high spatial resolution images acquired in the main growing season in 2010 or after 2010 were used for this task. Third, interpreters double-checked the selected forest and non-forest pixels with each other to guarantee the quality of these ground truth samples. Finally, 2397 forest pixels and 4330 non-forest pixels at the spatial resolution of 500 m were selected in monsoon Asia (Fig. 7). The accuracy assessment was carried out through confusion matrix in ENVI 5.2.

### Comparison with multiple forest cover datasets in 2010

We collected three remote sensing-based forest datasets (50-m JAXA F/NF, 300-m ESA F/NF, and 500-m MCD12Q1 F/NF), Landsat-based forest areas (>10% canopy), and inventory-based FAO FRA datasets available in the public domain for 2010. We compared the area and spatial distribution of these forest datasets in monsoon Asia at country and pixel scales. For the area comparison, we compared the forest areas from these forest maps in different countries. For the spatial comparison, we aggregated these forest maps into new forest maps at the spatial resolution of 1,500 m, the least common multiple of the spatial resolutions (50-m, 300-m and 500-m) from the collected forest datasets. This makes it convenient to compare the spatial differences among these forest datasets. We then analyzed the agreement and disagreement of these forest datasets through spatial overlay. The 500-m MODIS LSWI datasets in time series were used to distinguish evergreen and deciduous forests, which might result in mixed pixels with both evergreen and deciduous forests. Here, we use the approach put forward by Fritz *et al*.^59^,^60^, and the proposed approach takes into account all possible situations in which there is an overlap between land cover definitions. Supplementary Table S1 provides a brief introduction of these forest datasets for inter-comparison.

## Additional Information

**How to cite this article**: Qin, Y. *et al*. Mapping forests in monsoon Asia with ALOS PALSAR 50-m mosaic images and MODIS imagery in 2010. *Sci. Rep.* **6**, 20880; doi: 10.1038/srep20880 (2016).

## Supplementary Material

Supplementary Information

## References

[b1] Foley J. A. . Global consequences of land use. Science 309, 570–574, doi: 10.1126/science.1111772 (2005). 16040698

[b2] Bonan G. B. Forests and climate change: Forcings, feedbacks, and the climate benefits of forests. Science 320, 1444–1449, doi: 10.1126/science.1155121 (2008). 18556546

[b3] van der Werf G. R. . CO2 emissions from forest loss. Nat Geosci 2, 737–738, doi: 10.1038/ngeo671 (2009).

[b4] IPCC. Climate Change 2007: The Physical Science Basis. Contribution of Working Group I to the Fourth Assessment Report of the Intergovernmental Panel on Climate Change [ Solomon S., Qin D., Manning M., Chen Z., Marquis M., Averyt K. B., Tignor M. & Miller H. L. (eds)]. (Cambridge University Press, Cambridge, United Kingdom and New York, NY, USA, 996 pp., 2007).

[b5] DeFries R. . Earth observations for estimating greenhouse gas emissions from deforestation in developing countries. Environ Sci Policy 10, 385–394, doi: 10.1016/j.envsci.2007.01.010 (2007).

[b6] Grassi G., Monni S., Federici S., Achard F. & Mollicone D. Applying the conservativeness principle to REDD to deal with the uncertainties of the estimates. Environ Res Lett 3, doi: Artn 035005 doi: 10.1088/1748-9326/3/3/035005 (2008).

[b7] Achard F. & Hansen M. C. Global forest monitoring from earth observation. (CRC Press, 2012).

[b8] Margono B. A., Potapov P. V., Turubanova S., Stolle F. & Hansen M. C. Primary forest cover loss in Indonesia over 2000–2012. Nature Climate Change 4, 730–735, doi: 10.1038/Nclimate2277 (2014).

[b9] Stibig H. J., Achard F., Carboni S., Rasi R. & Miettinen J. Change in tropical forest cover of Southeast Asia from 1990 to 2010. Biogeosciences 11, 247–258, doi: 10.5194/bg-11-247-2014 (2014).

[b10] Yu G. . High carbon dioxide uptake by subtropical forest ecosystems in the East Asian monsoon region. Proc Natl Acad Sci USA 111, 4910–4915, doi: 10.1073/pnas.1317065111 (2014). 24639529 PMC3977309

[b11] Fang J. Y. . Forest biomass carbon sinks in East Asia, with special reference to the relative contributions of forest expansion and forest growth. Global Change Biol 20, 2019–2030, doi: 10.1111/Gcb.12512 (2014). 24464906

[b12] Hansen M. C. & DeFries R. S. Detecting long-term global forest change using continuous fields of tree-cover maps from 8-km advanced very high resolution radiometer (AVHRR) data for the years 1982–99. Ecosystems 7, 695–716, doi: 10.1007/s10021-004-0243-3 (2004).

[b13] Loveland T. R. . Development of a global land cover characteristics database and IGBP DISCover from 1 km AVHRR data. Int J Remote Sens 21, 1303–1330, doi: 10.1080/014311600210191 (2000).

[b14] Friedl M. A. . MODIS Collection 5 global land cover: Algorithm refinements and characterization of new datasets. Remote Sens Environ 114, 168–182, doi: 10.1016/j.rse.2009.08.016 (2010).

[b15] Hansen M. C., Shimabukuro Y. E., Potapov P. & Pittman K. Comparing annual MODIS and PRODES forest cover change data for advancing monitoring of Brazilian forest cover. Remote Sens Environ 112, 3784–3793, doi: 10.1016/j.rse.2008.05.012 (2008).

[b16] Hansen M. C. . High-Resolution Global Maps of 21st-Century Forest Cover Change. Science 342, 850–853, doi: 10.1126/science.1244693 (2013). 24233722

[b17] Kim D. H. . Global, Landsat-based forest-cover change from 1990 to 2000. Remote Sens Environ 155, 178–193, doi: 10.1016/j.rse.2014.08.017 (2014).

[b18] Shimada M. . New global forest/non-forest maps from ALOS PALSAR data (2007–2010). Remote Sens Environ 155, 13–31, doi: 10.1016/j.rse.2014.04.014 (2014).

[b19] Dong J. W. . A comparison of forest cover maps in Mainland Southeast Asia from multiple sources: PALSAR, MERIS, MODIS and FRA. Remote Sens Environ 127, 60–73, doi: 10.1016/j.rse.2012.08.022 (2012).

[b20] Blaes X., Vanhalle L. & Defourny P. Efficiency of crop identification based on optical and SAR image time series. Remote Sens Environ 96, 352–365, doi: 10.1016/j.rse.2005.03.010 (2005).

[b21] Corbane C., Faure J. F., Baghdadi N., Villeneuve N. & Petit M. Rapid Urban Mapping Using SAR/Optical Imagery Synergy. Sensors-Basel 8, 7125–7143, doi: 10.3390/S8117125 (2008). PMC3787435 27873921

[b22] Zhu Z., Woodcock C. E., Rogan J. & Kellndorfer J. Assessment of spectral, polarimetric, temporal, and spatial dimensions for urban and peri-urban land cover classification using Landsat and SAR data. Remote Sens Environ 117, 72–82, doi: 10.1016/j.rse.2011.07.020 (2012).

[b23] Xiao X. M., Biradar C. M., Czarnecki C., Alabi T. & Keller M. A Simple Algorithm for Large-Scale Mapping of Evergreen Forests in Tropical America, Africa and Asia. Remote Sens-Basel 1, 355–374, doi: 10.3390/Rs1030355 (2009).

[b24] Xiao X. M., Boles S., Liu J. Y., Zhuang D. F. & Liu M. L. Characterization of forest types in Northeastern China, using multi-temporal SPOT-4 VEGETATION sensor data. Remote Sens Environ 82, 335–348, doi: Pii S0034-4257(02)00051-2 doi: 10.1016/S0034-4257(02)00051-2 (2002).

[b25] Beck P. S. A., Atzberger C., Hogda K. A., Johansen B. & Skidmore A. K. Improved monitoring of vegetation dynamics at very high latitudes: A new method using MODIS NDVI. Remote Sens Environ 100, 321–334, doi: 10.1016/j.rse.2005.10.021 (2006).

[b26] Sexton J. O. . Conservation policy and the measurement of forests. Nature Climate Change advance online publication, doi: 10.1038/nclimate2816 (2015).

[b27] Wylie D., Jackson D. L., Menzel W. P. & Bates J. J. Trends in global cloud cover in two decades of HIRS observations. J Climate 18, 3021–3031, doi: 10.1175/Jcli3461.1 (2005).

[b28] European Space Agency. Land Cover CCI PRODUCT USER GUIDE (Version 2). (2014).

[b29] Asner G. P. . Selective logging in the Brazilian Amazon. Science 310, 480–482, doi: 10.1126/science.1118051 (2005). 16239474

[b30] Dong J. W. . A 50-m Forest Cover Map in Southeast Asia from ALOS/PALSAR and Its Application on Forest Fragmentation Assessment. Plos One 9, doi: ARTN e85801 doi: 10.1371/journal.pone.0085801 (2014). PMC3899076 24465714

[b31] Edwards D. P. . Degraded lands worth protecting: the biological importance of Southeast Asia’s repeatedly logged forests. P Roy Soc B-Biol Sci 278, 82–90, doi: 10.1098/rspb.2010.1062 (2011). PMC2992721 20685713

[b32] Curran L. M. . Lowland forest loss in protected areas of Indonesian Borneo. Science 303, 1000–1003, doi: 10.1126/science.1091714 (2004). 14963327

[b33] DeFries R., Hansen A., Newton A. C. & Hansen M. C. Increasing isolation of protected areas in tropical forests over the past twenty years. Ecol Appl 15, 19–26, doi: 10.1890/03-5258 (2005).

[b34] Sodhi N. S. . The state and conservation of Southeast Asian biodiversity. Biodivers Conserv 19, 317–328, doi: 10.1007/s10531-009-9607-5 (2010).

[b35] Meyfroidt P. & Lambin E. F. Forest transition in Vietnam and displacement of deforestation abroad. P Natl Acad Sci USA 106, 16139–16144, doi: 10.1073/pnas.0904942106 (2009). PMC2752536 19805270

[b36] Thuiller W., Araujo M. B. & Lavorel S. Do we need land-cover data to model species distributions in Europe? J Biogeogr 31, 353–361 (2004).

[b37] Feddema J. J. . The importance of land-cover change in simulating future climates. Science 310, 1674–1678, doi: 10.1126/science.1118160 (2005). 16339443

[b38] Miettinen J., Shi C. H. & Liew S. C. Deforestation rates in insular Southeast Asia between 2000 and 2010. Global Change Biol 17, 2261–2270, doi: 10.1111/j.1365-2486.2011.02398.x (2011).

[b39] Koh L. P., Miettinen J., Liew S. C. & Ghazoul J. Remotely sensed evidence of tropical peatland conversion to oil palm. P Natl Acad Sci USA 108, 5127–5132, doi: 10.1073/pnas.1018776108 (2011). PMC3064377 21383161

[b40] Penuelas J., Rutishauser T. & Filella I. Phenology Feedbacks on Climate Change. Science 324, 887–888, doi: 10.1126/science.1173004 (2009). 19443770

[b41] Dong J. W. . Mapping deciduous rubber plantations through integration of PALSAR and multi-temporal Landsat imagery. Remote Sens Environ 134, 392–402, doi: 10.1016/j.rse.2013.03.014 (2013).

[b42] FAO. *FAOSTAT. Emissions - Land Use*, < http://faostat3.fao.org/download/G2/*/E> (2013).

[b43] Shimada M., Isoguchi O., Tadono T. & Isono K. PALSAR Radiometric and Geometric Calibration. Ieee T Geosci Remote 47, 3915–3932, doi: 10.1109/Tgrs.2009.2023909 (2009).

[b44] Vermote E. F., Kotchenova S. Y. & Ray J. P. MODIS Surface Reflectance User’s Guide. (2011).

[b45] Xiao X. . Landscape-scale characterization of cropland in China using Vegetation and landsat TM images. Int J Remote Sens 23, 3579–3594, doi: 10.1080/01431160110106069 (2002).

[b46] Xiao X. . Observation of flooding and rice transplanting of paddy rice fields at the site to landscape scales in China using VEGETATION sensor data. Int J Remote Sens 23, 3009–3022, doi: 10.1080/01431160110107734 (2002).

[b47] Maki M., Ishiahra M. & Tamura M. Estimation of leaf water status to monitor the risk of forest fires by using remotely sensed data. Remote Sens Environ 90, 441–450, doi: 10.1016/j.rse.2004.02.002 (2004).

[b48] Food and Agriculture Organization of the United Nations. Global Forest Resource Assessment (FRA) 2010. (Rome, 2012).

[b49] Imhoff M. L. A Theoretical-Analysis of the Effect of Forest Structure on Synthetic-Aperture Radar Backscatter and the Remote-Sensing of Biomass. Ieee T Geosci Remote 33, 341–352, doi: 10.1109/36.377934 (1995).

[b50] Ni W. J. . Retrieval of Forest Biomass From ALOS PALSAR Data Using a Lookup Table Method. Ieee J-Stars 6, 875–886, doi: 10.1109/Jstars.2012.2212701 (2013).

[b51] Shimada M. . New global forest/non-forest maps from ALOS PALSAR data (2007-2010). Remote Sens Environ 155, 13–31, doi: 10.1016/j.rse.2014.04.014 (2014).

[b52] Peregon A. & Yamagata Y. The use of ALOS/PALSAR backscatter to estimate above-ground forest biomass: A case study in Western Siberia. Remote Sens Environ 137, 139–146, doi: 10.1016/j.rse.2013.06.012 (2013).

[b53] Yuan F., Sawaya K. E., Loeffelholz B. C. & Bauer M. E. Land cover classification and change analysis of the Twin Cities (Minnesota) Metropolitan Area by multitemporal Landsat remote sensing. Remote Sens Environ 98, 317–328, doi: 10.1016/j.rse.2005.08.006 (2005).

[b54] Defries R. S. & Townshend J. R. G. Ndvi-Derived Land-Cover Classifications at a Global-Scale. Int J Remote Sens 15, 3567–3586 (1994).

[b55] Lunetta R. S., Knight J. F., Ediriwickrema J., Lyon J. G. & Worthy L. D. Land-cover change detection using multi-temporal MODIS NDVI data. Remote Sens Environ 105, 142–154, doi: 10.1016/j.rse.2006.06.018 (2006).

[b56] Thenkabail P. S., Schull M. & Turral H. Ganges and Indus river basin land use/land cover (LULC) and irrigated area mapping using continuous streams of MODIS data. Remote Sens Environ 95, 317–341, doi: 10.1016/j.rse.2004.12.018 (2005).

[b57] Li Z. & Fox J. M. Mapping rubber tree growth in mainland Southeast Asia using time-series MODIS 250 m NDVI and statistical data. Appl Geogr 32, 420–432, doi: 10.1016/j.apgeog.2011.06.018 (2012).

[b58] Solano R., Didan K., Jacobson A. & Huete A. MODIS Vegetation Index User’s Guide (MOD13 Series). (Vegetation Index and Phenology Lab, The University of Arizona, 2010).

[b59] Fritz S. & See L. Identifying and quantifying uncertainty and spatial disagreement in the comparison of Global Land Cover for different applications. Global Change Biol 14, 1057–1075, doi: 10.1111/j.1365-2486.2007.01519.x (2008).

[b60] Fritz S. & Lee L. Comparison of land cover maps using fuzzy agreement. Int J Geogr Inf Sci 19, 787–807, doi: 10.1080/13658810500072020 (2005).

