# Supplementary material for: Mapping forests in monsoon Asia with ALOS PALSAR 50-m mosaic images and MODIS imagery in 2010
Source: Sci Rep. 2016 Feb 11;6:20880. doi: 10.1038/srep20880 (PMC4750005; doi:10.1038/srep20880)
Supplement: Supplementary Information [file srep20880-s1.pdf]

Supplementary Information

Mapping forests in monsoon Asia with ALOS PALSAR 50-m mosaic images and MODIS imagery in 2010

Yuanwei Qin, Xiangming Xiao, Jinwei Dong, Geli Zhang, Partha Sarathi Roy, Pawan Kumar Joshi, Hammad Gilani, Manchiraju Sri Ramachandra Murthy, Cui Jin, Jie Wang, Yao Zhang, Bangqian Chen, Michael Angelo Menarguez, Chandrashekhar M. Biradar, Rajen Bajgain, Xiangping Li, Shengqi Dai, Ying Hou, Fengfei Xin, Berrien Moore III

A. Supplementary Figure Legends and Figures

B. Supplementary Tables

A. Supplementary Figure Legends and Figures

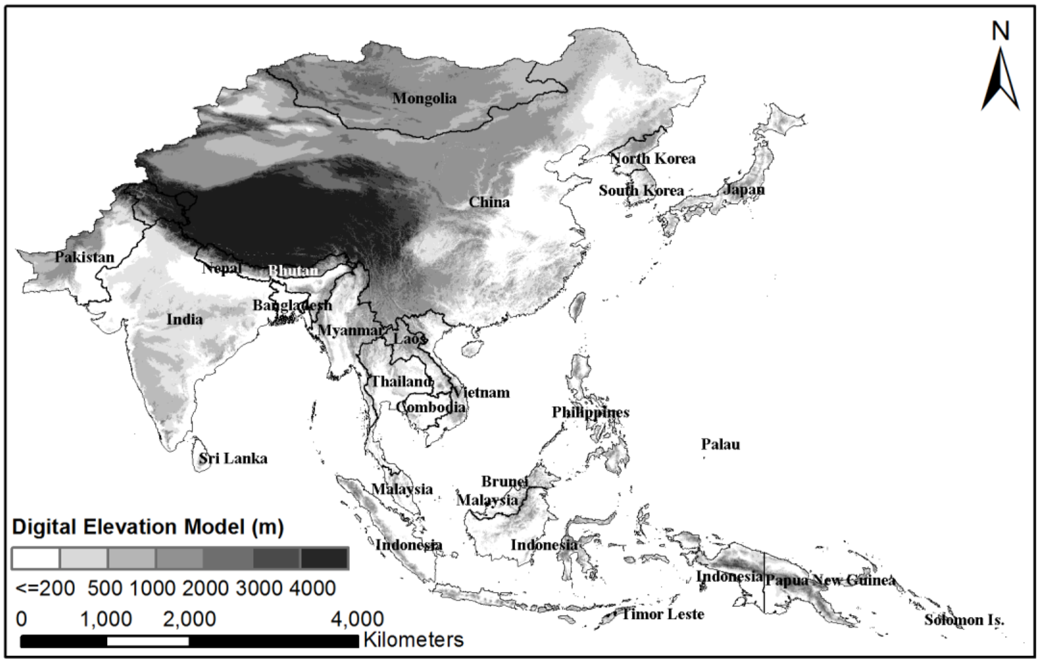

Figure S1. The study area of monsoon Asia. The background is the 1-km Digital Elevation Model (DEM) from Global 30 Arc-Second Elevation (GTOPO30) (https://lta.cr.usgs.gov/GTOPO30). The country boundaries come from the Food and Agriculture Organization of the United Nations (http://data.fao.org/map?entryId=025a52fb-639d-4547-a9aa-7d2a6de5aae0&tab=metadata). This figure was produced using ArcGIS 10.1.

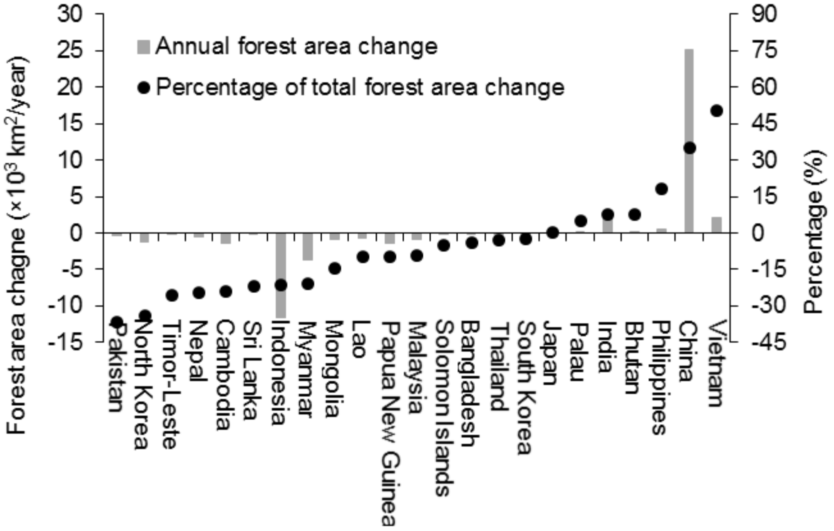

Figure S2. Forest area changes in Monsoon Asia from 1990 to 2012 and the data provided by FAOSTAT[1]. This figure was produced using Microsoft Excel 2013.

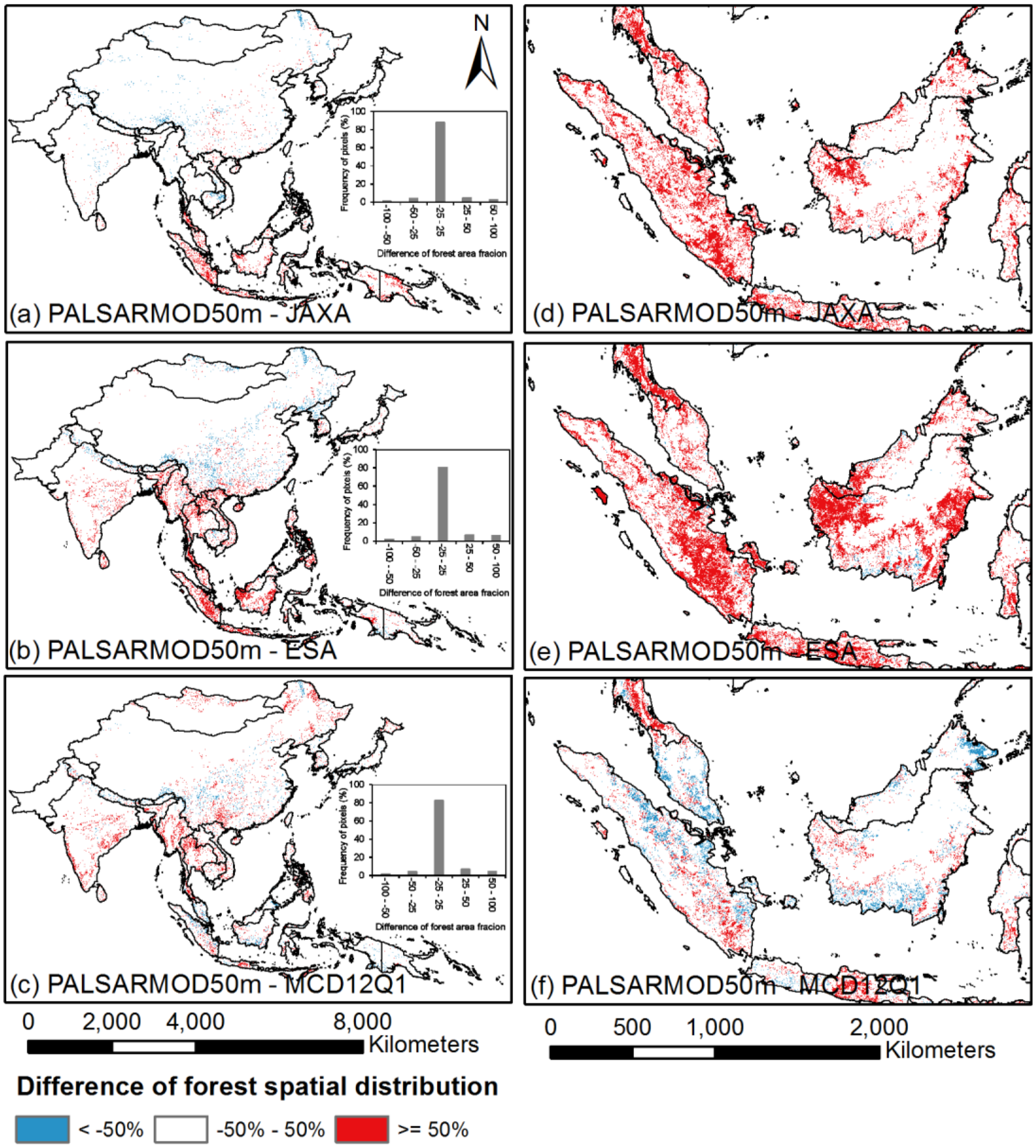

Figure S3. Spatial differences among multiple forest maps at the spatial resolution of 1,500 m in monsoon Asia in 2010. (**a**) Spatial difference between PALSARMOD50m forest/non-forest map and JAXA forest/non-forest map. (**b**) Spatial difference between PALSARMOD50m forest/non-forest map and ESA forest/non-forest map. (**c**) Spatial difference between PALSARMOD50m forest/non-forest map and MCD12Q1 forest/non-forest map. (**d, e, f**) Zoomed-in maps in Southeast Asia from (**a, b, c**), respectively. This figure was produced using ArcGIS 10.1.

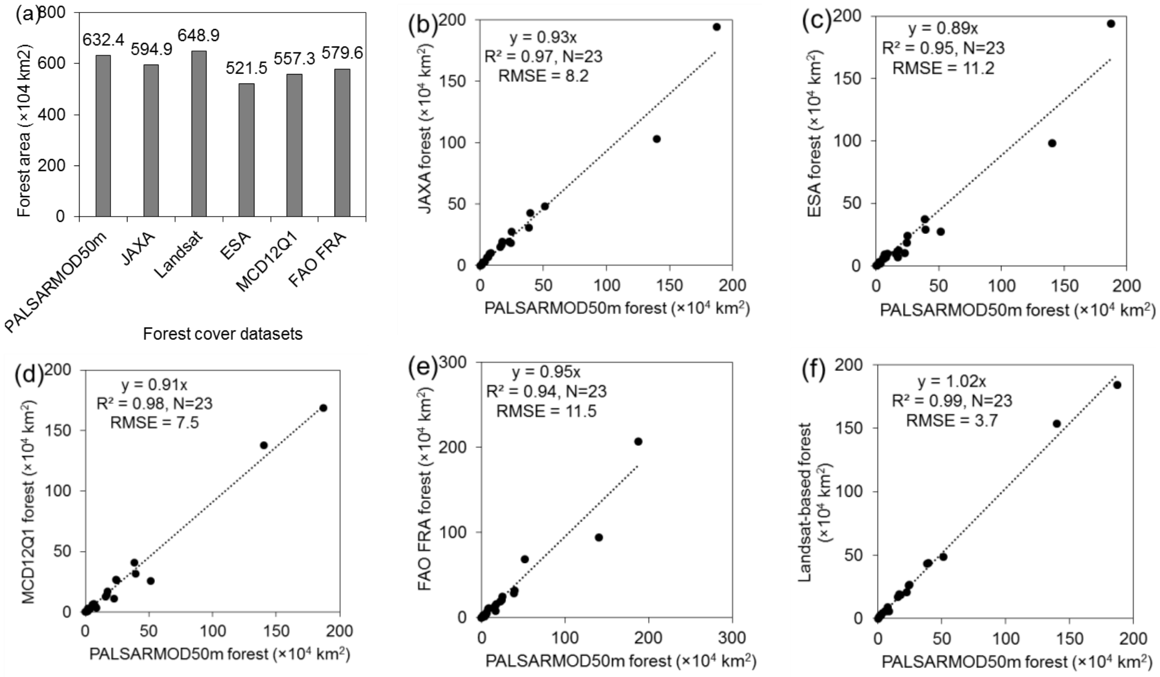

Figure S4. Forest area comparisons at the country scale from multiple forest datasets in monsoon Asia in 2010. (**a**) Forest areas from multiple forest datasets in monsoon Asia. (**b, c, d, e, f**) The linear relationships between PALSARMOD50m forest area and the forest areas from JAXA, ESA, MCD12Q1, FAO FRA, and Landsat-based forest datasets. This figure was produced using Microsoft Excel 2013.

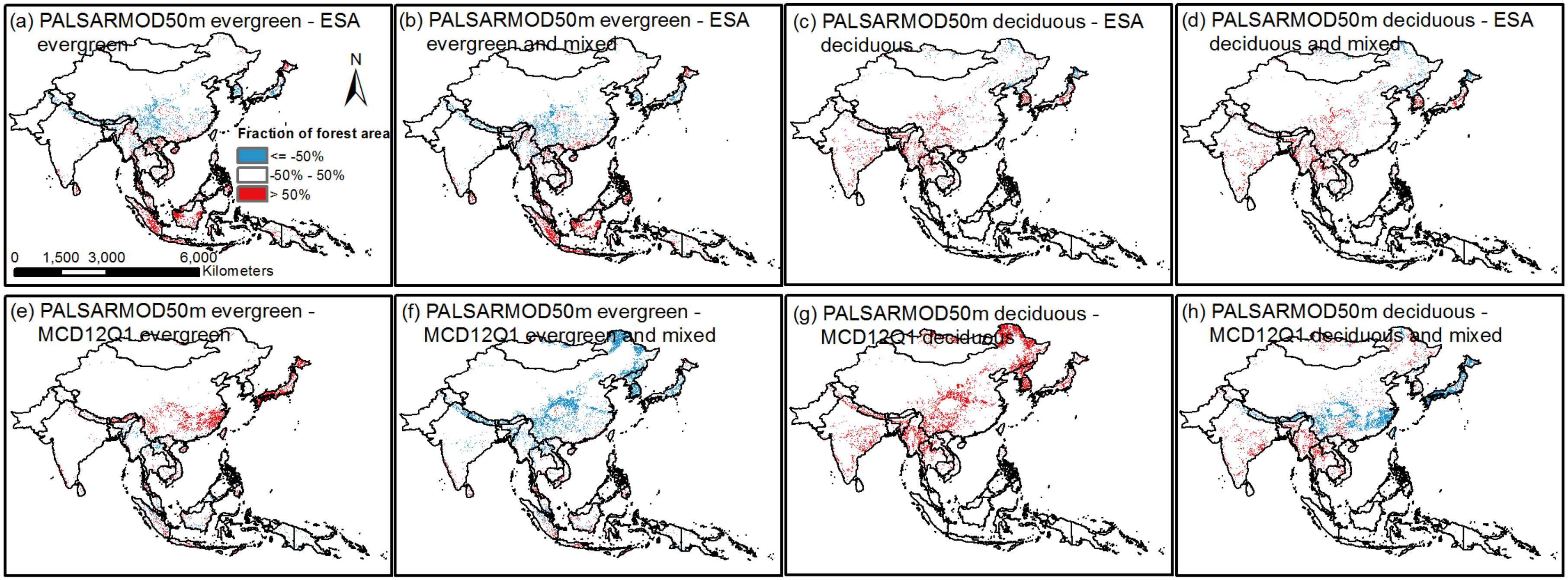

Figure S5. Spatial differences of evergreen and deciduous forests between PALSARMOD50m forest/non-forest and ESA, MCD12Q1 forest/non-forest maps at the spatial resolution of 1,500 in monsoon Asia. (**a, b**) Spatial differences between PALSARMOD50m evergreen forests and ESA evergreen forests, and evergreen and mixed forests, respectively. (**c, d**) Spatial differences between PALSARMOD50m deciduous forests and ESA deciduous forests, and deciduous and mixed forests, respectively. (**e, f**) Spatial differences between PALSARMOD50m evergreen forests and MCD12Q1 evergreen forests, and evergreen and mixed forests, respectively. (**g, h**) Spatial differences between PALSARMOD50m deciduous forests and MCD12Q1 deciduous forests, and deciduous and mixed forests, respectively. This figure was produced using ArcGIS 10.1.

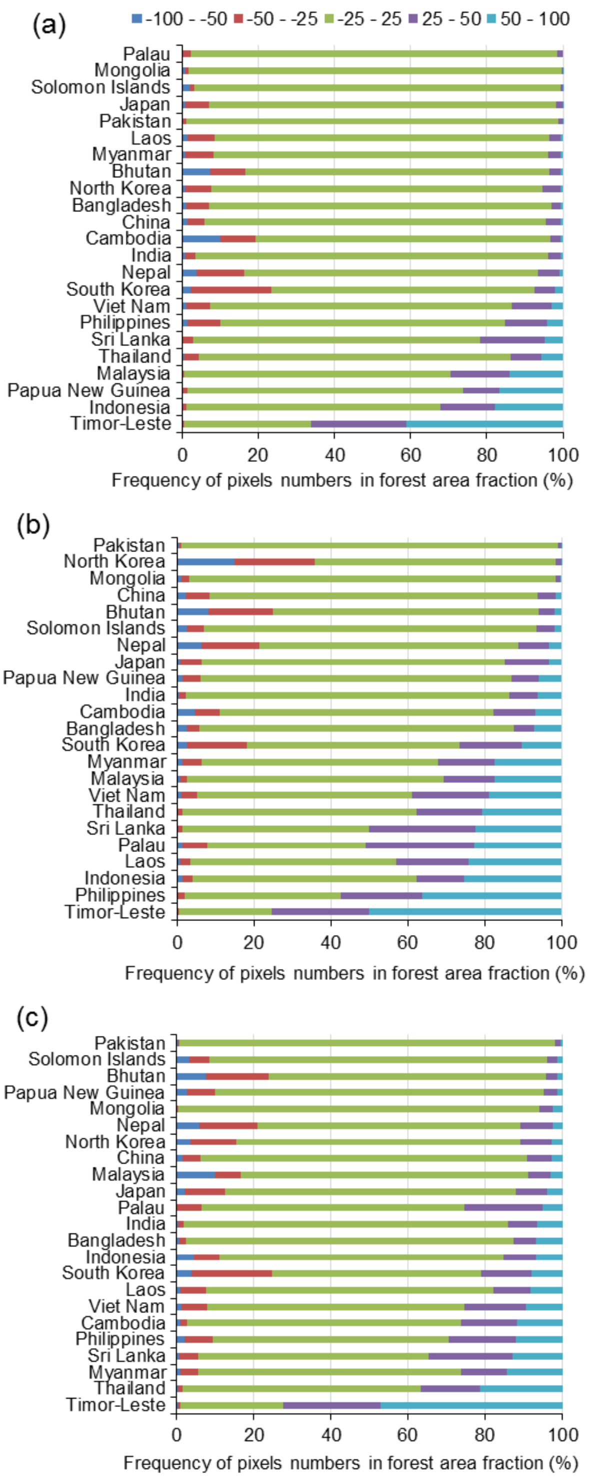

Figure S6. Comparison of the fraction of forest area at the spatial resolution of 1,500 m. (**a**) Comparison between PALSARMOD50m forest/non-forests map and JAXA forest/non-forest map, i.e., PALSARMOD50m –JAXA. (**b**) Comparison between PALSARMOD50m forest/non-forests map and ESA forest/non-forest map, i.e., PALSARMOD50m – ESA. (**c**) Comparison between PALSARMOD50m forest/non-forests map and MCD12Q1 forest/non-forest map, i.e., PALSARMOD50m – MCD12Q1. This figure was produced using Microsoft Excel 2013.

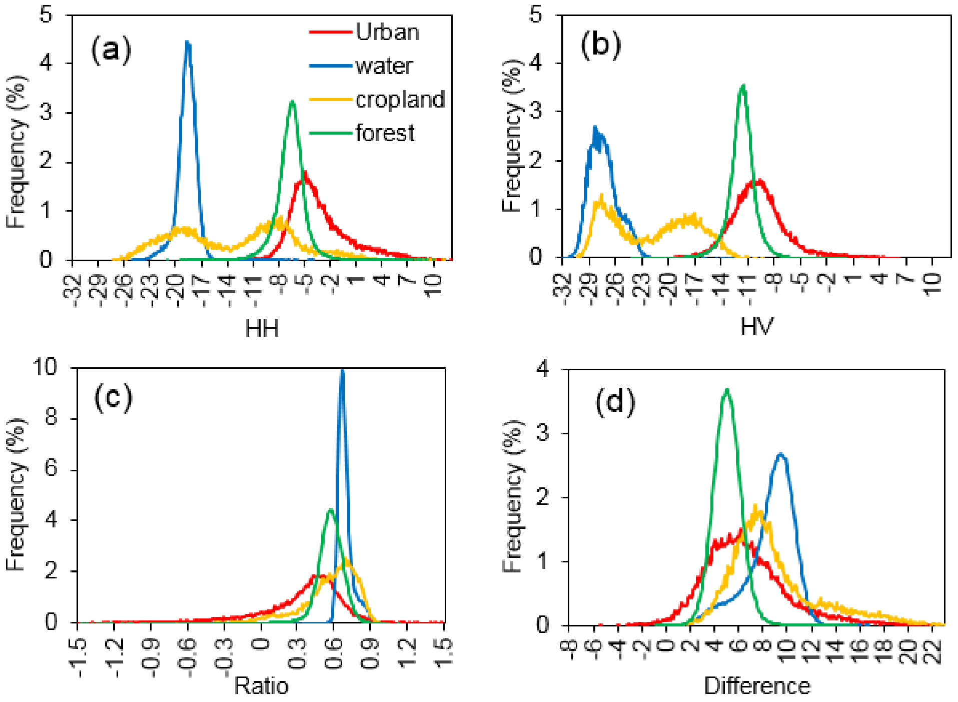

Figure S7. The backscatter signatures of different land cover types based on the ALOS PALSAR gamma naught. (**a, b, c, d**) are the HH, HV, Ratio (HH/HV), and Difference (HH-HV), respectively. This figure was produced using Microsoft Excel 2013.

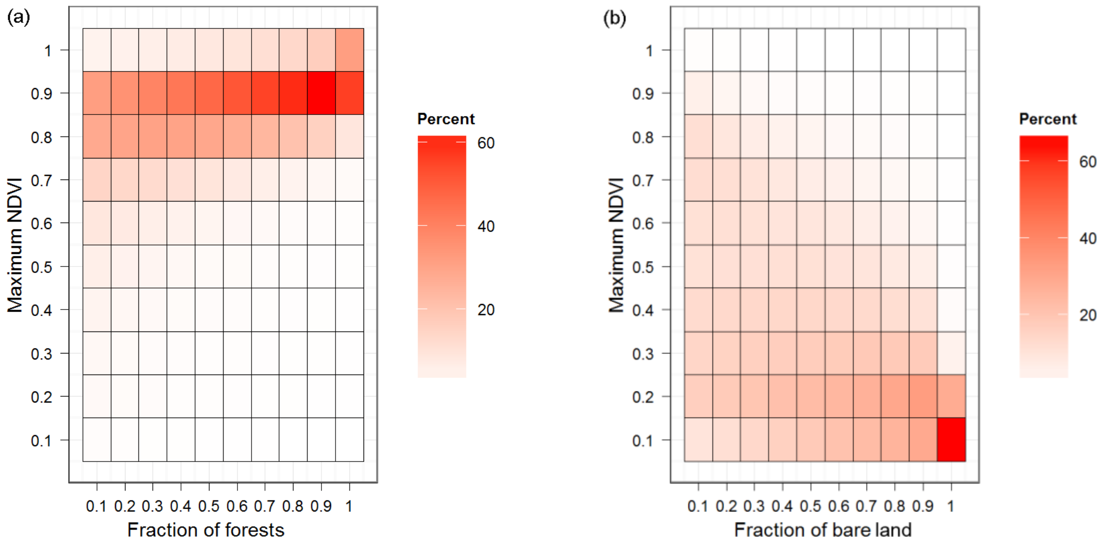

Figure S8. The frequency of the annual maximum NDVI of forest and bare land in different fractions. (**a**) Forest. (**b**) Bare land. NDVI data was from the 16-day MOD13Q1 NDVI product (250-m spatial resolution) for 2010. The 1-km forest and bare land layers were from the 2010 land use/cover map generated by the Chinese Academy of Sciences through the visual interpretation of Landsat TM/ETM+ images[2,3] (http://www.resdc.cn/data.aspx?DATAID=99). This figure was produced using R 3.2.0.

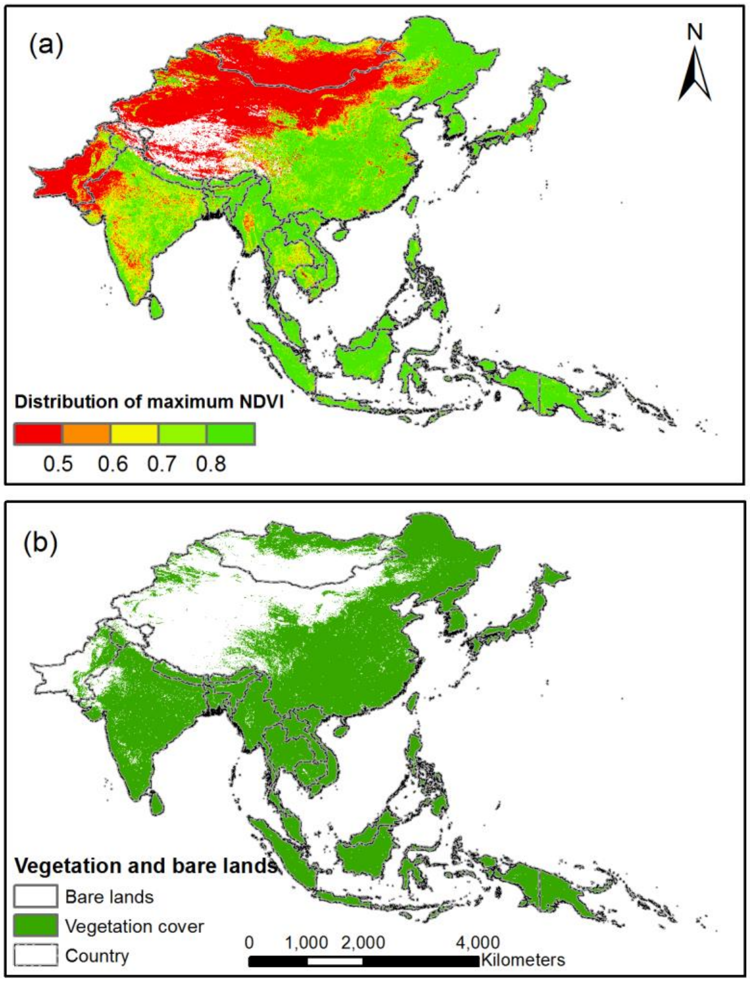

Figure S9. Spatial distribution of maximum NDVI and vegetation lands in monsoon Asia. (**a**) Maximum NDVI from 16-day MOD13Q1 NDVI product at the spatial resolution of 250 m in 2010. (**b**) Vegetation and bare lands maps generated from (**a**), based on the threshold value of maximum NDVI (0.5). This figure was produced using ArcGIS 10.1.

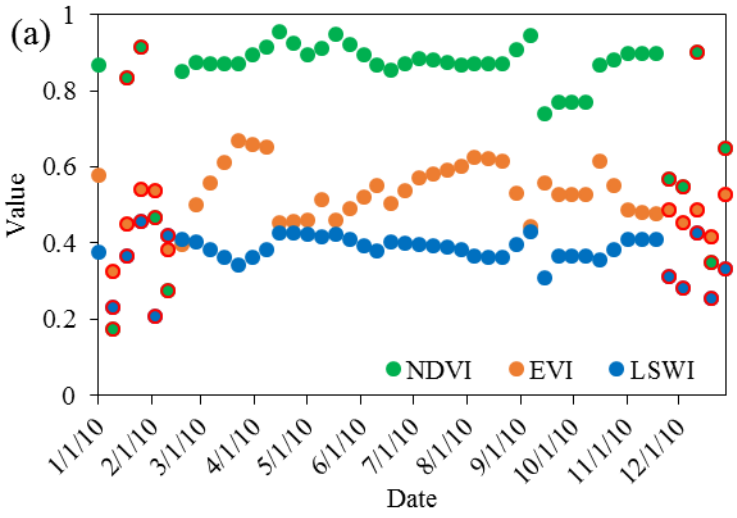

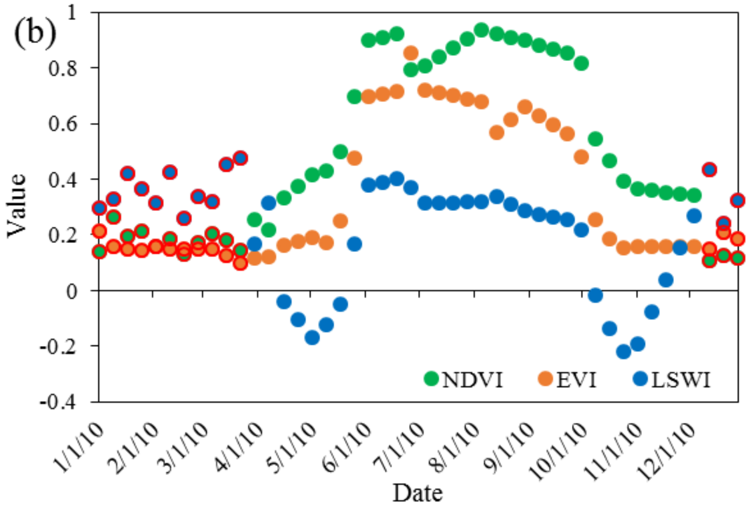

Figure S10. Temporal profiles of NDVI, EVI, and LSWI for evergreen and deciduous forests in monsoon Asia in 2010, derived from 8-day MOD09A1 products at the spatial resolution of 500 m. The time series of MOD09A1 products are available from the Earth Observation and Modeling Facility in the University of Oklahoma (http://www.eomf.ou.edu/). (**a**) NDVI, EVI, and LSWI for evergreen forest (0.8133°N, 109.6975°E); (**b**) NDVI, EVI, and LSWI of deciduous forest (41.2862°N, 124.7911°E). The gaps caused by three or less continuous bad observations (cloud, cloud shadows, snow/ice, et al.) were filled through linear interpolation. Those gaps caused by four or more continuous bad observations were not processed and were labeled with red circles. This figure was produced using Microsoft Excel 2013.

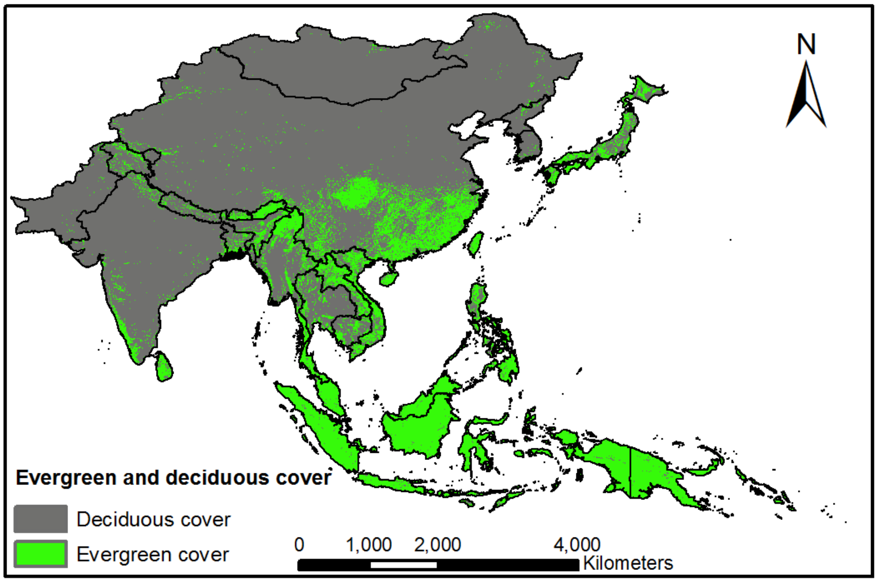

Figure S11. Spatial distribution map of evergreen and deciduous vegetation in monsoon Asia in 2010 based on the 8-day Land Surface Water Index (LSWI) at a spatial resolution of 500 m derived from MOD09A1 land surface reflectance products. This figure was produced using ArcGIS 10.1.

B. Supplementary Tables

Table S1. Brief description of the multiple forest datasets in 2010 used in this study.

Forest cover datasets	Forest land cover types	Spatial resolution	Algorithms	Data source	References
MCD12Q1 (IGBP)	Woody vegetation with a percent coverage over 60% and tree height exceeding 2-m.	500-m	Supervised classification	500-m aggregated 32-day average nadir BRDF-adjusted reflectance (NBAR), enhanced vegetation index (EVI), Land Surface Temperature (LST), and annual metrics (min, max, and mean values) for EVI, LST and NBAR bands in 2010	[4,5]
ESA CCI-LC	Woody vegetation with a percent coverage over 15%.	300-m	Unsupervised classification	300-m land cover map produced by 7-day time series MERIS imagery during 2003-2012 as baseline, 1-km SPOT-VEGETATION (2008-2012) time series for updating	[6]
JAXA F/NF	Woody vegetation coverage over 10%, determined by high spatial resolution images in Google Earth.	50-m	Supervised classification	25-m PALSAR Fine Beam Double Polarization mode data from June to September in 2010	[7]
PALSARMOD50m F/NF	Woody vegetation coverage over 10% determined by high spatial resolution images in Google Earth.	50-m	Supervised classification	50-m PALSAR Fine Beam Double Polarization mode data from June to September in 2010	This study
FAO FRA	Land spanning over 0.5 ha with tree height exceeding 5-m and a canopy cover more than 10%, or trees able to reach these thresholds in situ.	Country	Statistical datasets	Country statistics in 2010	[8]
Landsat forest	Canopy closure with a percent coverage over 10% and all vegetation taller than 5-m in height.	Country	Supervised classification	30-m Landsat ETM+ images in growing season in circa 2010	[9]

Table S2. The confusion matrix between the PALSAR/MODIS-based forest map and validation samples in the Areas of Interest (AOIs) collected through high resolution images in Google Earth and geo-referenced photos from the fields.

Class		Ground truth samples (pixels)		Total classified pixels	User accuracy (%)	Commission error (%)
		**Forest**	Non-forest			
Classification	**Forest**	211729	2396	214125	98.88	1.12
	Non-forest	27898	492485	520383	94.64	5.36
Total ground truth pixels		239627	494881	734508		
Producer accuracy (%)		88.36	99.52			
Omission error (%)		11.64	0.48			
Overall accuracy (%)		95.88		Kappa coefficient = 0.90		

Table S3. Forest areas from PALSARMOD50m, JAXA, Landsat (the University of Maryland), MCD12Q1, ESA, and FAO FRA forest datasets at country scale in monsoon Asia in 2010.

Country	PALSARMOD50m F/NF			JAXA	Landsat	MCD12Q1 F/NF				ESA F/NF				FAO FRA
	Total	Evergreen	Deciduous			Total	Evergreen	Deciduous	Mixed	Total	Evergreen	Deciduous	Mixed	
Bangladesh	1.97	0.84	1.12	2.07	2.60	0.77	0.40	0.01	0.37	1.06	0.27	0.80	0.00	1.44
Bhutan	2.27	1.75	0.53	2.71	2.67	2.82	0.96	0.01	1.85	2.78	2.65	0.13	0.00	3.25
Cambodia	7.67	4.97	2.68	9.34	9.29	5.04	4.75	0.07	0.24	6.80	4.54	2.27	0.00	10.09
China	187.33	67.86	119.46	194.50	184.11	168.60	21.17	9.49	137.89	194.01	100.17	92.42	1.42	206.86
North Korea	6.48	0.45	6.02	6.78	5.74	6.93	0.04	1.28	5.60	9.03	0.44	8.53	0.05	5.67
India	51.47	14.04	37.45	48.43	48.39	25.82	11.14	2.94	11.74	27.15	14.81	12.38	0.00	68.43
Indonesia	140.12	138.68	1.29	103.27	153.33	137.73	136.89	0.11	0.56	98.13	97.86	0.00	0.27	94.43
Japan	24.89	17.60	7.25	27.31	26.93	26.25	0.76	2.09	23.26	24.11	17.22	6.48	0.41	24.98
Laos	17.65	13.74	3.88	19.14	18.81	16.87	16.73	0.05	0.15	12.21	11.17	1.06	0.00	15.75
Malaysia	24.26	24.09	0.17	18.36	26.04	26.83	26.76	0.01	0.02	18.71	18.69	0.00	0.02	20.46
Mongolia	8.72	0.53	8.13	10.01	5.77	3.65	0.19	0.14	3.34	9.77	0.52	9.20	0.04	10.90
Myanmar	39.55	22.54	16.97	42.88	43.89	31.70	24.73	0.67	6.35	28.90	22.05	6.86	0.00	31.77
Nepal	5.52	1.38	4.15	6.41	5.57	6.32	0.08	0.01	6.24	6.30	4.11	2.20	0.00	3.64
Pakistan	1.49	0.25	1.24	1.18	1.62	0.67	0.42	0.00	0.24	1.26	0.89	0.36	0.00	1.69
Palau	0.03	0.03	0.00	0.03	0.04	0.03	0.02	0.00	0.00	0.02	0.02	0.00	0.00	0.04
Papua New Guinea	38.72	38.56	0.14	31.00	43.20	40.90	40.62	0.01	0.24	37.47	37.39	0.00	0.07	28.73
Philippines	16.98	15.36	1.63	16.43	19.27	14.16	13.79	0.16	0.12	6.81	6.76	0.00	0.05	7.67
South Korea	6.04	0.38	5.64	7.04	5.67	6.26	0.07	0.92	5.24	5.64	3.01	2.61	0.02	6.22
Solomon Islands	2.30	2.29	0.00	2.44	2.70	2.45	2.41	0.00	0.01	2.40	2.36	0.00	0.05	2.21
Sri Lanka	3.53	3.21	0.32	2.83	4.17	2.48	2.42	0.02	0.04	1.67	1.64	0.03	0.00	1.86
Thailand	22.62	12.00	10.61	19.30	21.01	10.79	9.84	0.12	0.80	10.30	7.77	2.55	0.00	18.97
Timor-Leste	0.90	0.76	0.14	0.30	0.83	0.25	0.22	0.01	0.02	0.20	0.20	0.00	0.00	0.74
Vietnam	15.82	12.67	3.17	14.89	17.26	12.84	11.99	0.10	0.61	9.36	8.53	0.80	0.04	13.80
Total	632.44	398.36	233.76	594.78	648.91	557.34	329.40	18.23	209.10	521.51	369.79	149.35	2.47	579.59

1 FAO. *FAOSTAT. Emissions - Land Use*, <http://faostat3.fao.org/download/G2/*/E> (2013).

2 Liu, J. Y. *et al.* Spatiotemporal characteristics, patterns, and causes of land-use changes in China since the late 1980s. *J Geogr Sci* **24**, 195-210, doi:10.1007/s11442-014-1082-6 (2014).

3 Zhang, Z. X. *et al.* A 2010 update of National Land Use/Cover Database of China at 1:100000 scale using medium spatial resolution satellite images. *Remote Sens Environ* **149**, 142-154, doi:DOI 10.1016/j.rse.2014.04.004 (2014).

4 Friedl, M. A. *et al.* MODIS Collection 5 global land cover: Algorithm refinements and characterization of new datasets. *Remote Sens Environ* **114**, 168-182, doi:DOI 10.1016/j.rse.2009.08.016 (2010).

5 Friedl, M. A. *et al.* Global land cover mapping from MODIS: algorithms and early results. *Remote Sens Environ* **83**, 287-302, doi:Doi 10.1016/S0034-4257(02)00078-0 (2002).

6 European Space Agency. Land Cover CCI PRODUCT USER GUIDE (Version 2). (2014).

7 Shimada, M. *et al.* New global forest/non-forest maps from ALOS PALSAR data (2007–2010). *Remote Sens Environ* **155**, 13-31, doi:10.1016/j.rse.2014.04.014 (2014).

8 Nations, F. a. A. O. o. t. U. Global Forest Resource Assessment (FRA) 2010. (Rome, 2012).

9 Hansen, M. C. *et al.* High-Resolution Global Maps of 21st-Century Forest Cover Change. *Science* **342**, 850-853, doi:DOI 10.1126/science.1244693 (2013).

107
